# Dysbiosis Anticipating Necrotizing Enterocolitis in Very Premature Infants

**DOI:** 10.1093/cid/ciu822

**Published:** 2014-10-23

**Authors:** Kathleen Sim, Alexander G. Shaw, Paul Randell, Michael J. Cox, Zoë E. McClure, Ming-Shi Li, Munther Haddad, Paul R. Langford, William O. C. M. Cookson, Miriam F. Moffatt, J. Simon Kroll

**Affiliations:** 1 Department of Medicine, Section of Paediatrics; 2 Department of Molecular Genetics and Genomics, National Heart and Lung Institute; 3 Department of Paediatric Surgery, Imperial College London, United Kingdom

**Keywords:** necrotizing enterocolitis, NEC, fecal microbiota, premature infant

## Abstract

***Background.*** Necrotizing enterocolitis (NEC) is a devastating inflammatory bowel disease of premature infants speculatively associated with infection. Suspected NEC can be indistinguishable from sepsis, and in established cases an infant may die within hours of diagnosis. Present treatment is supportive. A means of presymptomatic diagnosis is urgently needed. We aimed to identify microbial signatures in the gastrointestinal microbiota preceding NEC diagnosis in premature infants.

***Methods.*** Fecal samples and clinical data were collected from a 2-year cohort of 369 premature neonates. Next-generation sequencing of 16S ribosomal RNA gene regions was used to characterize the microbiota of prediagnosis fecal samples from 12 neonates with NEC, 8 with suspected NEC, and 44 controls. Logistic regression was used to determine clinical characteristics and operational taxonomic units (OTUs) discriminating cases from controls. Samples were cultured and isolates identified using matrix-assisted laser desorption/ionization–time of flight. Clostridial isolates were typed and toxin genes detected.

***Results.*** A clostridial OTU was overabundant in prediagnosis samples from infants with established NEC (*P* = .006). Culture confirmed the presence of *Clostridium perfringens* type A. Fluorescent amplified fragment-length polymorphism typing established that no isolates were identical. Prediagnosis samples from NEC infants not carrying profuse *C. perfringens* revealed an overabundance of a *Klebsiella* OTU (*P* = .049). Prolonged continuous positive airway pressure (CPAP) therapy with supplemental oxygen was also associated with increased NEC risk.

***Conclusions.*** Two fecal microbiota signatures (*Clostridium* and *Klebsiella* OTUs) and need for prolonged CPAP oxygen signal increased risk of NEC in presymptomatic infants. These biomarkers will assist development of a screening tool to allow very early diagnosis of NEC.

***Clinical Trials Registration.*** NCT01102738.

## Abstract (precis)

Using 16S rRNA gene sequencing and targeted culture, we compared microbiota in fecal samples from infants with necrotizing enterocolitis (NEC) and controls. Two significant signatures were associated with NEC: 1 with dominant *Clostridium perfringens* and 1 with dominant Enterobacteriaceae.

Necrotizing enterocolitis (NEC) is a devastating disease primarily affecting premature infants, with an incidence increasing with lower gestational age. Remarkable progress in neonatal intensive care over the past 2 decades has enabled the survival of increasingly premature, vulnerable babies, but with this comes an increase in cases of NEC [1]. NEC has a high mortality—up to 35% in extremely low-birth-weight infants (<1000 g) [2]—and in survivors, gastrointestinal (GI) dysfunction and functional impairment may be lifelong [3].

A strategy for surveillance/screening is essential to reduce the impact of this condition, for the timely diagnosis of NEC is difficult. Using Bell staging [4], the earliest signs (Bell stage 1 [suspected NEC]) are indistinguishable from sepsis (bloodstream infection). In cases when NEC has been confirmed (Bell stages 2 [definite] and 3 [severe]), clinical deterioration may be so rapid that the infant dies within hours of diagnosis. Although the Bell staging system is useful for grouping signs and symptoms, it does not address etiology [5]. Despite >1.5 centuries of research [6], the cause of this inflammatory bowel disease remains elusive, and treatment strategies remain largely supportive and nonspecific.

NEC is thought to be a multifactorial disease requiring an immature gut, enteral feeds, and bacterial colonization for its development [7]. Evidence supporting the contribution of an infectious agent stems from well-documented outbreaks [8]; an association between empirical use of antibiotics in the first week of life and an increased risk of NEC [9], possibly due to the potential distortion of the GI microbiota; and the observation that NEC does not occur in a gnotobiotic setting [10]. Toll-like receptor 4 expression on enterocytes is necessary for disease to occur [11], and may explain why its onset is inversely proportional to gestational age at birth. No single organism has been consistently implicated as causing NEC, and it is thought that abnormal colonization of the GI tract may play a role in pathogenesis [7]. This has suggested various probiotic interventions as possible prophylactic strategies [12].

To capture the evolution of GI microbiota from birth in the relatively small number of infants who develop NEC, we collected almost every fecal sample and >100 fields of daily clinical data from every baby born at a 2-site tertiary neonatal unit over a 2-year period. We used the culture-independent approach afforded by next-generation sequencing to characterize microbes on the basis of variation in the domains of the 16S ribosomal RNA (rRNA) gene. Combined with additional methods, we identified 2 significant signatures associated with future NEC: 1 characterized by an apparent bloom of *Clostridium perfringens*, and the other dominated by a *Klebsiella* operational taxonomic unit (OTU).

## METHODS

### Study Population

Infants born before 32 completed weeks of gestation admitted to an Imperial College Healthcare National Health Service Trust neonatal intensive care unit (NICU) (St Mary's Hospital, Queen Charlotte's and Chelsea Hospital) between January 2010 and December 2012 were eligible for inclusion in the study unless considered to be in extremis in the first days of life. The NICU is a tertiary center with approximately 700 admissions per annum. Of the 388 eligible infants, 369 were recruited.

Both hospitals have identical feeding, antibiotic, and antifungal protocols, and staff members rotate between sites. Probiotics and H_2_ receptor antagonists are not used on the unit. Detailed daily clinical information was collected from the patients' records.

### Ethics Declaration

The study “Defining the Intestinal Microbiota in Premature Infants” (ClinicalTrials.gov identifier NCT01102738) was approved by West London Research Ethics Committee Two, United Kingdom (reference number 10/H0711/39). Parents gave written informed consent for their infant to participate in the study.

### Sample Collection

We collected almost every fecal sample produced by each participant from the point of recruitment until discharge. Samples were collected by nursing staff from diapers using a sterile spatula, placed in a sterile DNAase-, RNAase-free Eppendorf tube, stored in a −20°C freezer on the neonatal unit within 2 hours of collection, and stored at −80°C within 5 days.

### Case Definition, Control Selection, and Clinical Management

An NEC case was defined using the Vermont Oxford Network criteria [13] and staged according to the Bell modified staging criteria [14]. The diagnosis was made by the attending neonatal consultant and confirmed by an independent neonatologist. Three control infants (no NEC or bloodstream infection/sepsis diagnosis during admission) were selected for each NEC Bell stage 2/3 case based on postnatal age. Where possible, control infants were subsequently matched by mode of delivery, admission hospital, and antibiotic use, as these factors are known to influence the microbiota [15–18]. Infants with suspected NEC were similarly paired to a single control infant. As a separate substudy, each infant with NEC (Bell stage 1, 2, and 3) was independently matched to a contemporaneous control on the same site to address the possibility of an outbreak. Investigators were not involved in clinical care.

### Bacterial DNA Extraction

Fecal samples (200 mg) were processed using the FastDNA SPIN Kit for Soil (MP Biomedicals), incorporating a bead-beating step for mechanical disruption of cells. We have established that this effectively lyses gram-positive and -negative bacteria in fecal samples (unpublished data). Extractions were performed following the manufacturer's protocol except that the final elution step was into Tris (10 mM) low-ethylenediaminetetraacetic acid (0.1 mM) buffer.

### Polymerase Chain Reaction Amplification and Pyrosequencing of the V3–V5 Regions of the Bacterial 16S rRNA Gene

The V3–V5 [19] region of bacterial 16S rRNA genes was amplified from each DNA sample using a primer pair tagged with individually unique 12-bp error-correcting Golay barcodes [20, 21]. Polymerase chain reaction (PCR) was performed as previously described, except that the reaction was cycled 35 times to maximize amplification from samples of low DNA concentration [21]. Replicate amplicons were pooled and purified, and 4 pyrosequencing runs were carried out on a 454 Life Sciences GS FLX (Roche) following the Roche Amplicon Lib-L protocol. Replicate samples were spread over all sequencing runs as internal controls.

### Bioinformatics

Shotgun processed data were denoised using AmpliconNoise [22] as part of the Quantitative Insights Into Microbial Ecology version 1.5.0 package [23], followed by chimera removal with ChimeraSlayer [24]. Sequences were aligned using the SILVA rRNA database (SSU_REF108) [25] for reference and clustered at 97% sequence identity using the UCLUST algorithm [26] into OTUs. Representative sequences were selected and classified using the Ribosomal Database Project Classifier [27]. Rarefaction was performed, removing heterogeneity of sequencing reads per sample.

### Statistical Analysis

Case and control patient characteristics were compared using Student's *t* tests and χ^2^ tests or Fisher's exact test where appropriate. OTUs that were differentially abundant between NEC and control groups were identified using logistic regression performed using the generalized linear model and stepwise algorithm of the R statistical package (version 3.0.2) [28]. Comparisons of OTU count data were performed using Student's *t* tests (unequal variances). *P* values <.05 were considered to be significant.

### Data Availability

16S rRNA amplicon data have been deposited at the European Nucleotide Archive under accession number PRJEB6345.

### Bacterial Culture and Identification

An antecedent frozen sample as close as possible to the day of diagnosis of NEC (D_0_) from every case and their corresponding contemporaneous control were selected for culture of both *Clostridium* species and Enterobacteriaceae. For isolation of *Clostridium* species, an alcohol shock method [29] was used to eliminate non-spore-forming organisms, and the suspension plated on Fastidious Anaerobic Agar (Oxoid). Additionally, samples were inoculated on to CHROMagar (BD) to aid identification of Enterobacteriaceae. All bacterial isolates were identified by matrix-assisted laser desorption/ionization–time of flight using a Bruker Microflex LT (Bruker Daltonics).

### Typing and Toxin Multiplexed PCR

Isolates of *C. perfringens* were analyzed at the Public Health England Foodborne Pathogens reference service for screening for the presence of alpha, beta, epsilon, iota, cpe, and beta2toxin genes using multiplexed PCR. Fluorescent amplified fragment-length polymorphism (fAFLP) typing was undertaken to establish (non)-relatedness of isolates.

## RESULTS

### Patients

**Figure 1.**
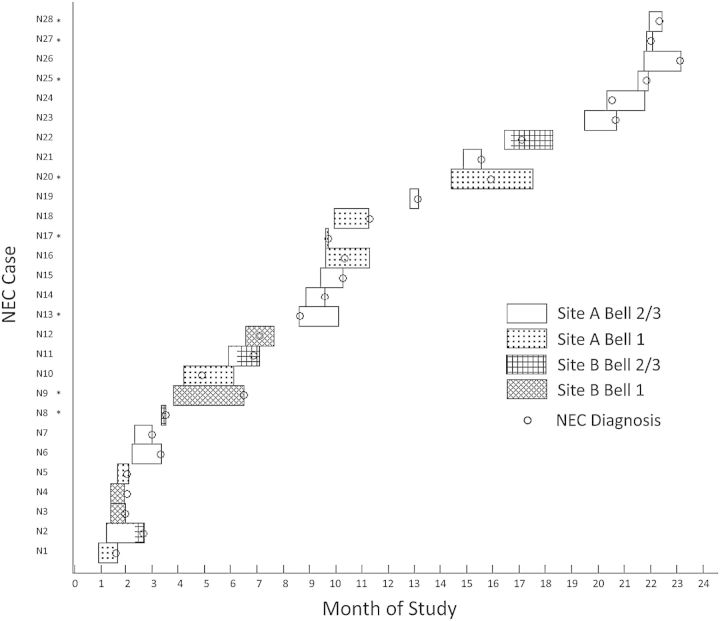
Cumulative cases of necrotizing enterocolitis (NEC) over 24 months. Hatching indicates hospital site and Bell staging. Bar length indicates duration of infant admission on the neonatal unit and circles indicate date of NEC diagnosis. Asterisked cases were excluded from the analyses due to lack of samples (n = 2), sequencing data (n = 4), or controls (n = 2).

Three hundred sixty-nine infants were recruited and 10 928 fecal samples collected. Twenty-eight infants developed NEC during the study period: 10 with Bell stage 1, 2 with Bell stage 2, and 16 with Bell stage 3. Figure 1 illustrates the distribution of the cases over the 2 years. Six infants with Bell stage 2/3 were not included in analysis due to lack of samples (developed NEC prior to obtaining consent) (n = 2), or inadequate sequencing reads (n = 4). Two infants with Bell stage 1 could not be included due to lack of controls. Table 1 outlines the cohort clinical characteristics of Bell stage 2/3 infants (12 cases analyzed, 6 not included in analysis) and controls. Comparisons are displayed between the 12 cases analyzed and, respectively, the 6 not included in analysis, and controls. Control infants turned out to have significantly more days not requiring respiratory support, and a different birth hospital, from cases. There were no demographic differences such as mean birth weight, gestation at birth, birth hospital, mode of delivery, or antibiotic use between the Bell stage 2/3 infants who were and were not included in the analysis. Through their developing NEC prior to obtaining consent, the average age at diagnosis was lower in the infants who were not included in the analysis.

**Table 1.** Summary of Cohort Characteristics: Cases With Necrotizing Enterocolitis Bell Stage 2/3 and Controls

Characteristic	Cases Analyzed (n = 12)	Cases Not Analyzed (n = 6)	Controls (n = 36)
Demographics			
Male	5 (41.7)	3 (50.0)	17 (47.2)
Mean birth weight (IQR), g	845.4 (685.0–898.8)	899.3 (732.5–1033.3)	1005.9 (755.0–1239.5)
Mean gestation at birth (IQR), weeks + days	27 + 0 (25 + 5–28 + 3)	27 + 1 (26 + 5–28 + 1)	27 + 3 (25 + 3–29 + 0)
Mean postnatal age at D_0_ (IQR), d	27.5 (20.8–37.5)*	7.2 (4.5–10.0)*	NA
Mean postnatal age at sample taken closest to D_0_ (IQR), d	26.0 (19–36.3)	NA	24.4 (18.0–34.8)
Birth hospital			
Site A	11 (91.7)**	5 (83.3)	21 (58.3)**
Site B	1 (8.3)**	0 (0)	7 (19.4)**
Other	0 (0.0)**	1 (17.7)	8 (22.2)**
Ethnicity			
Black	2 (16.7)	2 (33.3)	8 (22.2)
White	5 (41.7)	2 (33.3)	18 (50.0)
Asian	2 (16.7)	1 (16.7)	6 (16.7)
Mixed race	2 (16.7)	1 (16.7)	3 (8.3)
Unknown	1 (8.3)	0 (0)	1 (2.8)
Mode of delivery			
Cesarean	7 (58.3)	3 (50.0)	18 (50.0)
Vaginal	5 (41.7)	3 (50.0)	18 (50.0)
Maternal characteristics			
Maternal IVAB use at delivery	2 (16.7)	2 (33.3)	11 (30.6)
Maternal PROM	2 (16.7)	2 (33.3)	9 (25.0)
Maternal sepsis/chorioamnionitis	3 (25.0)	2 (33.3)	6 (16.7)
Condition at birth			
Mean lowest pH recorded^a^ in the first 24 h (IQR)	7.2 (7.2–7.3)	7.2 (7.1–7.2)	7.2 (7.1–7.3)
Mean lowest base excess recorded^a^ in the first 24 h, (IQR)	−5.5 (−6.8 to −2.65)	−7.4 (−8.8 to −4.9)	−6.3 (−9.1 to −3.5)
Mean APGAR at 5 min (IQR)	7.8 (7.0–9.0)	8.2 (8.0–9.0)	7.4 (7.0–9.0)
Intravenous antibiotic use			
Mean No. of d of IVAB^b^ during first week of life (IQR)	2.1 (0.8–3.0)	2.2 (0.5–2.8)	2.6 (2.0–3.0)
Mean No. of cumulative d of IVAB^b^ use prior to D_0_ (IQR)	3.7 (1.2–3)	2.8 (2.0–3.8)	3.6 (2.0–4.0)
Feeding regime			
Mean No. of d received MEBM prior to D_0_ (IQR)	22.8 (15.0–30.8)	4.0 (2.3–6.0)***	25.3 (18.0–33.0)
Mean No. of d received DEBM prior to D_0_ (IQR)	11.9 (7.5–13.3)	3.7 (0.25–5.5)***	7.8 (4.0–9.0)
Mean No. of d received formula milk prior to D_0_ (IQR)	0.7 (0–0)	1.0 (0–0)	0.4 (0–0)
Respiratory support requirement			
Mean No. of d requiring ventilation support (HFOV or conventional ventilation) prior to D_0_ (IQR)	1.8 (0–2.3)	3.8 (2.0–4.5)	3.3 (0–2.8)
Mean No. of d requiring CPAP (no oxygen), prior to D_0_ (IQR)	7.2 (1–10.8)	1.5 (0–2.8)***	8.2 (0–13.8)
Mean No. of d requiring CPAP with supplemental oxygen, prior to D_0_ (IQR)	16.8 (1.8–26.5)	1.8 (0–2.3)***	8.2 (0–13.8)
Mean No. of d requiring NP oxygen, prior to D_0_ (IQR)	1.1 (0–0)	0 (0–0)	2.6 (0–4.0)
Mean No. of d not requiring any respiratory support, prior to D_0_ (IQR)	0.7 (0–0)	0 (0–0)	4.8 (4.0–10.1)
Presence of PDA	2 (16.7)	2 (33.3)	8 (22.2)

Data are presented as No. (%) unless otherwise specified.

Abbreviations: APGAR, Appearance, Pulse, Grimace, Activity, Respiration; CPAP, continuous positive airway pressure; DEBM, donor-expressed breast milk; D_0_, day of NEC diagnosis or postnatal age of matched control; HFOV, high-frequency oscillation ventilation; IQR, interquartile range; IVAB, intravenous antibiotics; MEBM, maternally expressed breast milk; NA, not applicable; NP, nasal prong; PDA, patent ductus arteriosus; PROM, prolonged rupture of membranes.

^a^ Lowest value recorded from cord/free flowing capillary/arterial/venous blood gas.

^b^ First-line IVAB for suspected early-onset sepsis: co-amoxiclav, or benzylpenicillin and gentamicin (if ex utero transfer); second-line IVAB: vancomycin and piperacillin-tazobactam.

* *P* = .0001.

** *P* = .0001.

*** *P* < .05: confounded by infants with necrotizing enterocolitis (NEC) not included in the analysis systematically developing NEC at a significantly younger postnatal age.

### 454-Pyrosequencing and Initial Data Processing

At least 1 fecal sample per week from the 44 control infants was sequenced to determine the normal GI microbiota. From each NEC infant, weekly fecal samples from birth and 8 fecal samples collected in the 2 weeks (where possible) prior to D_0_ were characterized. No reduction in defecation frequency was observed in cases prior to D_0_. There were 174 and 369 samples successfully processed from NEC infants and controls, respectively. After denoising and chimera removal, 2 671 225 reads remained and were de-multiplexed. The mean number of reads was 4919 per sample. Sample reads were rarefied to 1000, with rarefaction curves showing this to be sufficient to capture the OTU diversity, as measured by the Shannon diversity index (Supplementary Figure 1). Singletons (sequences present only once in the dataset) and OTUs present in only 1 sample were removed.

### Bacterial Communities in Control Samples

**Figure 2.**
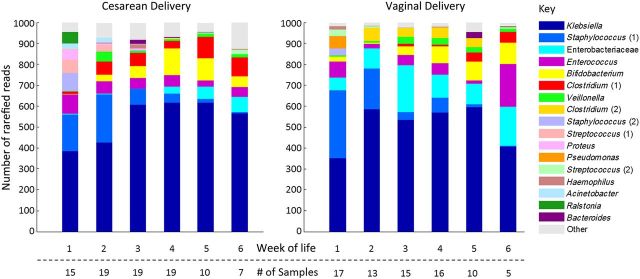
Bacterial community structure in control infants by postnatal week and mode of delivery. Data were generated using 1 sample per week from control infants using mean number of reads. When the same descriptive label (genus, family) is attached to >1 operational taxonomic unit (OTU; defined by 97% sequence similarity), these are numbered sequentially; no OTUs are combined.

The most abundant OTU descriptors were *Klebsiella*, *Staphylococcus* (1), Enterobacteriaceae, *Enterococcus*, and *Bifidobacterium*. Figure 2 shows the breakdown of OTUs grouped by postnatal week and mode of delivery. To determine whether the factors of day of sampling, delivery mode, and birth hospital needed to be controlled for, logistic regression was used to test for associations, allowing all 3 variables to be potential factors. Five OTUs were found to be correlated over time; there was an increase in *Bifidobacterium* and *Klebsiella* (*P* = .009 and *P* = .02, respectively), and a decrease in both *Staphylococcus* (1) and *Staphylococcus* (2) and *Streptococcus* (1) (*P* < .0001, *P* = .03, and *P* = .04, respectively). Vaginal delivery was found to be associated with Enterobacteriaceae and *Clostridium* (2) OTUs (*P* = .0009 and *P* = .04, respectively) and cesarean delivery with *Clostridium* (1) (*P* = .02). A weaker association was also found between the Enterobacteriaceae OTU and birth outside sites A and B (*P* = .05). See Supplementary Figure 2 for complete sequencing data.

### Bacterial Communities in NEC Samples

**Figure 3.**
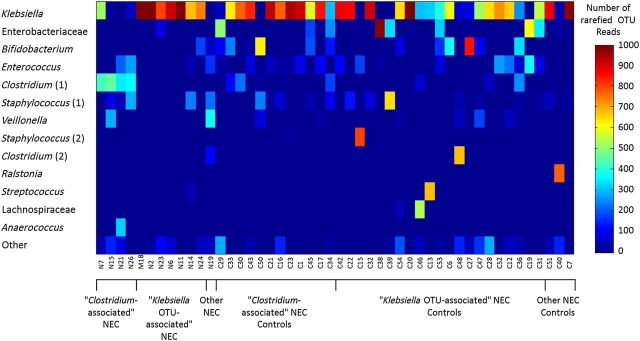
Bacterial communities in necrotizing enterocolitis (NEC) infant samples collected closest to the day of diagnosis of NEC (D_0_), and their matched controls. Samples are categorized along the x-axis. Color intensity indicates the number of rarefied reads from each operational taxonomic unit (OTU) that is found in a sample, as shown by the colored bar. Mean bacterial community diversity was not found to differ significantly between NEC and control infant samples.

In line with other published studies, our main analyses were performed on confirmed cases of NEC (Bell stage 2/3); suspected NEC cases were analyzed separately. The most abundant OTUs in samples collected closest to D_0_ in NEC Bell stage 2/3 cases (n = 12) were *Klebsiella*, *Clostridium* (1), *Staphylococcus* (1), and *Enterococcus* (Figure 3). We sought differences in OTU abundance of these samples compared with corresponding controls (n = 36) using a linear model to correct for clinical confounders. Models were constructed for each of the top 13 OTUs (95% of the dataset reads) to determine whether clinical factors shown in Table 1 and/or case/control status of the samples correlate with read numbers. After correcting for confounding factors, the OTU *Clostridium* (1) was significantly associated with cases (*P* = .006) (mean = 135 reads in cases, 26 in controls). High levels (350 reads) of this OTU were also found in 1 of the 8 suspected NEC infants, and 1 of their controls.

### Logistic Regression Analysis

Logistic regression was used to identify factors discriminating between the 12 Bell stage 2/3 case samples and the 36 control samples.

By univariate analysis including as variables the top 13 OTUs and a range of clinical characteristics (Table 1), we identified the number of *Clostridium* (1) OTU reads and days of continuous positive airway pressure (CPAP) oxygen as significant discriminators (*Clostridium* (1) OTU reads, based on the mean number of reads [135] in the NEC Bell stage 2/3 infants prior to diagnosis: odds ratio [OR], 2.5 [95% confidence interval {CI}, 1.2–5.2], *P* = .02; days of CPAP oxygen, based on a 7-day requirement in cases: OR, 1.5 [95% CI, 1.1–2.3], *P* = .04). In a multivariate analysis including all clinical variables, OTUs were added in turn, interaction with clinical factors was allowed, and the single OTU most improving the model was retained. Iteration with further OTUs was repeated until the model no longer improved.

The resulting model consisted of 4 terms, 2 of which were significant. The first significant term was the number of *Clostridium* (1) OTU reads, with an adjusted OR of 6.2 (95% CI, 1.8–21.7; *P* = .004). The second significant term was an interaction between the number of *Klebsiella* OTU reads and number of days the infant required CPAP oxygen prior to diagnosis. Based on the mean number of reads (559) found in the NEC Bell stage 2/3 infants and a 7-day requirement of CPAP oxygen, the adjusted OR was 4.6 (95% CI, 1.2–17.0; *P* = .02).

**Figure 4.**
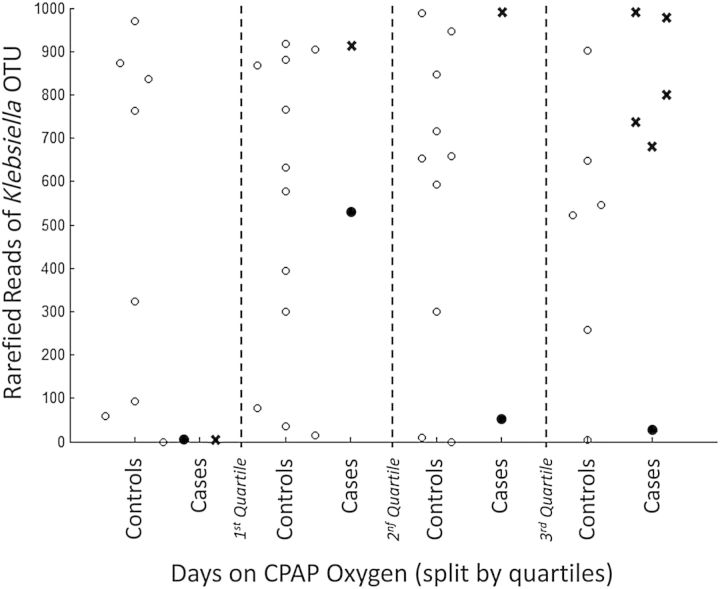
Relationship between *Klebsiella* operational taxonomic unit (OTU) reads and days of continuous positive airway pressure (CPAP) oxygen requirement by quartiles. Black circles indicate “*Clostridium*-associated” necrotizing enterocolitis (NEC); black X's indicate “*Klebsiella* OTU-associated” NEC. Control (white circles) and “*Clostridium*-associated” NEC samples are evenly spread over the quartiles. In contrast, “*Klebsiella* OTU-associated” NEC samples cluster above the third quartile.

**Figure 5.**
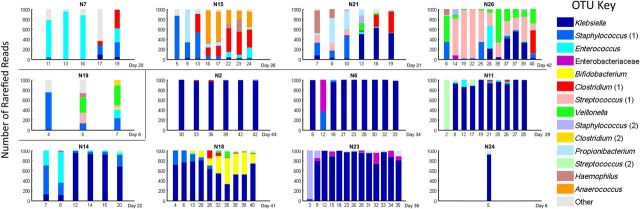
Evolution of the fecal microbiota in infants with necrotizing enterocolitis (NEC) Bell stage 2/3. The day of NEC diagnosis is shown at the bottom right of each bar chart. Cases defined as “*Clostridium*-associated” NEC are N7, N15, N21, and N26. Cases defined as “*Klebsiella* OTU-associated” NEC are N2, N6, N11, N14, N18, N23, and N24. N19 does not fit into either group. Abbreviation: OTU, operational taxonomic unit.

The relationship of the interactive term is explored in Figure 4. The risk factor associated with the *Clostridium* (1) OTU reads is shown to be independent of days requiring CPAP oxygen, reflected in the model by remaining an individual term. Cases were therefore defined according to which term contributed most strongly to indicate NEC, resulting in 4 cases of “*Clostridium-*associated” NEC and 7 of “*Klebsiella* OTU-associated” NEC. In these 7 cases, the samples closest to diagnosis had a higher abundance of *Klebsiella* OTU reads (mean = 870) than did control samples matched to these cases (mean = 450) (*P* < .0001). This dichotomy is seen in Figure 5 where the groups of cases display very different time courses. The final case (N19) satisfied neither criterion. We found no evidence for different NEC syndromes in our cohort of “*Clostridium*-associated” and “*Klebsiella* OTU-associated” patients.

### NEC Screening Criteria

Using the 2 significant factors above, a risk score for each of the NEC dichotomies was calculated for all samples from the 36 controls and the samples prior to diagnosis for the 12 cases. Calculated thresholds allowed correct classification of 10 of 12 cases and 25 of 36 control infants (sensitivity 0.83, specificity 0.69). The misclassified cases both occurred in the first week of life. The “*Clostridium*-associated” NEC cases were determined to be at risk an average of 5 days (standard deviation [SD], 5 days) prior to diagnosis, and “*Klebsiella* OTU-associated” NEC cases an average of 12 days (SD, 6 days) days prior to diagnosis. These presymptomatic high risk periods are reflected in the raw OTU data; the *Clostridium* (1) OTU was seen to increase significantly in the week preceding diagnosis (*P* = .004) in case samples, whereas the *Klebsiella* OTU remained at high levels in the 2 weeks prior to diagnosis.

These screening criteria were then tested on the Bell stage 1 infants, an extremely heterogenous group (only 1 of the babies went on to develop confirmed NEC). Two of 8 of the Bell stage 1 infants were classified as cases, and 1 control infant was misclassified.

### Identification of *Clostridium* Species

In all samples where the *Clostridium* (1) OTU was present (as at least 1% of sequencing reads), *C. perfringens* was isolated by culture. Although all *C. perfringens* isolates were type A, they all differed from one another by fAFLP typing. Three of the 6 isolates from infants with NEC were additionally found to contain the beta2 toxin gene. This was not found in any of the isolates from the 3 control infants (Supplementary Table).

### Identification of *Klebsiella* OTU Members

Although pyrosequencing indicated a predominant *Klebsiella* OTU, which was often the only Enterobacteriaceae OTU found, there was no single predominant species isolated on culture, with *Klebsiella pneumoniae*, *K. oxytoca*, *Enterobacter cloacae*, *Enterobacter aerogenes*, *Escherichia coli*, and *Serratia marcescens* all being identified. There was no systematic difference between samples from cases and controls.

## DISCUSSION

Previous studies have focused on identifying single causative pathogens in NEC, but none have been consistently found. Here we have demonstrated the presence of 2 separate groups of NEC infants based on the predominating organisms in the microbiota prior to diagnosis: *C. perfringens* or the *Klebsiella* OTU of Enterobacteriaceae. The microbial signature was the sole discriminating feature; these were not distinct clinical syndromes. Despite the well-documented clustering of NEC cases [8]—and there are temporally associated cases in our own cohort (eg, months 2, 9–10, and 20–22) (Figure 1)—our study refutes the proposition that any of these cases represented outbreaks. There is a mixture of “*Clostridium*-associated” NEC and “*Klebsiella* OTU-associated” NEC within each temporally associated group of cases, and every *C. perfringens* strain is distinct.

*Clostridium perfringens* has been considered as a putative etiological pathogen since NEC was first described. In animal models of NEC, *C. perfringens* isolated from fecal samples from premature infants with NEC cause cecal lesions [30]. In a clinical study, babies with NEC and *C. perfringens* in their stool had an earlier onset, more severe clinical course, larger extent of gangrene, and higher mortality rate [31]. The presence of strains bearing the plasmid-encoded beta2 toxin gene in 3 of the “*Clostridium*-associated” NEC cases, but none of the controls carrying low levels of *C. perfringens*, is intriguing. The toxin was originally characterized in a *C. perfringens* isolate from a piglet with necrotic enteritis [32], and has been shown to be cytotoxic for human colorectal epithelial (CaCo-2) cells [33]. More research is required to determine whether the beta2 toxin gene is expressed and contributes to NEC.

Our results from the “*Klebsiella* OTU-associated” NEC infants are consistent with recent NEC microbiota studies that have found an increase in the proportion of Enterobacteriaceae and other Proteobacteria in fecal samples taken from infants around the time of diagnosis [16, 34, 35]. Torrazza et al report a strong association between a *Klebsiella* OTU and NEC risk [16]. From samples containing a similarly labeled OTU in our study, we isolated multiple different species of Enterobacteriaceae, but could not associate NEC with an individual species. This highlights the necessity to go beyond 16S rRNA characterization, particularly when exploring the Enterobacteriaceae family, where the gene is relatively well conserved [36].

Persistently high levels of facultatively anaerobic Enterobacteriaceae in fecal samples are considered to represent a delay in maturation of the GI microbiota, along with a reduced level of anaerobes such as *Bifidobacterium* and *Bacteroides* [37]. Normally, with time, the GI microenvironment becomes depleted of oxygen, leading to a predominance of anaerobic organisms [38]. We speculate that prolonged need for CPAP oxygen days in our “*Klebsiella* OTU-associated” NEC infants may have affected the aerobicity of the gut lumen, allowing organisms such as Enterobacteriaceae to flourish.

This study focused on characterization of the fecal microbiota associated with NEC and does not address the mechanism of disease. In light of our findings, future in vitro work looking at toxin production of the *C. perfringens* isolates and potential virulence factors in the Enterobacteriaceae organisms may contribute toward understanding the pathophysiology. An important limitation of the study is that babies who develop NEC in the first week or so of life are systematically less likely to have provided sequential samples for analysis, and so were underrepresented. We might, however, speculate that NEC developing in these infants is less directly the consequence of the GI microbiota, rather reflecting some other complication of early extrauterine life. Of note, case N19, who did not fit into either the “*Clostridium*-associated” or “*Klebsiella* OTU-associated” NEC groups, developed NEC on day 8 of life.

The 2 microbial signatures identified in this study constitute potential biomarkers on which to base quantitative PCR surveillance of fecal samples to identify infants at high risk of NEC, while the ability to distinguish between “*Clostridium*-associated” NEC and “*Klebsiella* OTU-associated” NEC may allow early, specific treatment.

## Supplementary Data

Supplementary materials are available at *Clinical Infectious Diseases* online (http://cid.oxfordjournals.org). Supplementary materials consist of data provided by the author that are published to benefit the reader. The posted materials are not copyedited. The contents of all supplementary data are the sole responsibility of the authors. Questions or messages regarding errors should be addressed to the author.

Supplementary Data
