# Supplementary material for: Dysbiosis Anticipating Necrotizing Enterocolitis in Very Premature Infants
Source: Clin Infect Dis. 2014 Oct 23;60(3):389–97. doi: 10.1093/cid/ciu822 (PMC4415053; doi:10.1093/cid/ciu822)
Supplement: Supplementary Data [file supp_60_3_389__index.html]

Dysbiosis Anticipating Necrotizing Enterocolitis in Very Premature Infants — Dysbiosis Anticipating Necrotizing Enterocolitis in Very Premature Infants — Supplementary Data 

# Dysbiosis Anticipating Necrotizing Enterocolitis in Very Premature Infants

## Supplementary Data

Supplementary Data

**Files in this Data Supplement:**

- Supplementary Figure 1 - docx file
- Supplementary Figure 2 - docx file
- Supplementary Table - docx file
