# Supplementary material for: Dysbiosis Anticipating Necrotizing Enterocolitis in Very Premature Infants
Source: Clin Infect Dis. 2014 Oct 23;60(3):389–97. doi: 10.1093/cid/ciu822 (PMC4415053; doi:10.1093/cid/ciu822)
Supplement: Supplementary Data [file supp_ciu822_ciu822supp_fig1.docx]

**Supplementary Figure 1**

**Rarefaction curves for sequencing data.** A randomised selection of 10 % of the rarefaction curves for the dataset. Black dashed line shows the chosen cut off value for rarefaction.
