# Supplementary material for: Dysbiosis Anticipating Necrotizing Enterocolitis in Very Premature Infants
Source: Clin Infect Dis. 2014 Oct 23;60(3):389–97. doi: 10.1093/cid/ciu822 (PMC4415053; doi:10.1093/cid/ciu822)
Supplement: Supplementary Data [file supp_ciu822_ciu822supp_fig2.docx]

**Supplementary Figure 2**

**The GI microbiota of 44 control infants.** Samples are categorised along the x axis, grouped by infant and then chronologically with the earliest sample on the left. Infants are grouped by site. Colour intensity indicates the number of rarefied reads from each OTU that are found in a sample, as shown by the coloured bar.
