# Supplementary material for: Dysbiosis Anticipating Necrotizing Enterocolitis in Very Premature Infants
Source: Clin Infect Dis. 2014 Oct 23;60(3):389–97. doi: 10.1093/cid/ciu822 (PMC4415053; doi:10.1093/cid/ciu822)
Supplement: Supplementary Data [file supp_ciu822_ciu822supp_table.docx]

**Supplementary Table - *C. perfringens* toxin genes and fAFLP type.**

| Patient ID | Bell stage | Alpha toxin gene present | Beta2 toxin gene present | fAFLP^a^ type |
| --- | --- | --- | --- | --- |
| N3 | 1 | ✓ | ✓ | CLP.51 |
| N7 | 3 | ✓ |  | CLP.55 |
| N15^b^ | 3 | ✓ | ✓ | CLP.58 |
| N18^c^ | 2 | ✓ |  | CLP.52 |
| N21 | 3 | ✓ |  | CLP.54 |
| N26 | 3 | ✓ | ✓ | CLP.56 |
| C50^b^ | - | ✓ |  | CLP.57 |
| C52 | - | ✓ |  | CLP.60 |
| C17^c^ | - | ✓ |  | CLP.53 |

^a^ Fluorescent Amplified Fragment Length Polymorphism

^b,c^ case/control pairs
